# High Sensitivity C-Reactive Protein Predictive Value for Cardiovascular Disease: A Nested Case Control from Isfahan Cohort Study (ICS)

**DOI:** 10.5334/gh.367

**Published:** 2020-02-06

**Authors:** Pooya Koosha, Hamidreza Roohafza, Nizal Sarrafzadegan, Mehrbod Vakhshoori, Mohammad Talaei, Erfan Sheikhbahaei, Masoumeh Sadeghi

**Affiliations:** 1 Pediatric Cardiovascular Research Center, Cardiovascular Research Institute, Isfahan University of Medical Sciences, Isfahan, IR; 2 Psychosomatic Research Center, Isfahan University of Medical Sciences, Isfahan, IR; 3 Isfahan Cardiovascular Research Center, Cardiovascular Research Institute, Isfahan University of Medical Sciences, Isfahan, IR; 4 Heart Failure Research Center, Isfahan Cardiovascular Research Institute, Isfahan University of Medical Sciences, Isfahan, IR; 5 Institute of Population Health Sciences, Barts and the London School of Medicine, Queen Mary University of London, GB; 6 Interventional Cardiology Research Center, Cardiovascular Research Institute, Isfahan University of Medical Sciences, Isfahan, IR; 7 Cardiac Rehabilitation Research Center, Cardiovascular Research Institute, Isfahan University of Medical Sciences, Isfahan, IR

**Keywords:** high sensitivity C – reactive protein, cardiovascular events, inflammation, Isfahan cohort study, Odds ratio

## Abstract

**Background:**

High sensitivity C-reactive protein (hs-CRP) was proven to be an independent risk factor for cardiovascular diseases (CVDs). The aim of this study was to investigate the benefits of assessing hs-CRP among individuals with different cardiovascular risk factors.

**Methods:**

This nested case-control study was obtained from the Isfahan Cohort Study (ICS). Anyone who has been suffering from any CVDs, including myocardial infarction, unstable angina, sudden cardiac death and stroke was put in the case group. Density sampling method was utilized to choose the control group who had no aforementioned CVDs during follow-up. Four quartiles of hs-CRP (Q1: 0.1–2.3, Q2: 2.4–3, Q3: 3.1–4 and Q4: 4.1–14 mg/l) were assessed defining odds ratios (OR) of CVDs prediction in different CVDs risk factor categories. Confidence intervals of 95% are put in brackets.

**Results:**

A total of 502 cases and 538 controls were recruited. All hs-CRP quartiles showed increased CVDs likelihood compared to normal subjects in terms of diabetes mellitus (DM) and hypertension (HTN). Second quartile showed a 1.93 [1.33–2.81] and 3.34 [1.36–8.17] increased risks in patients with hypertriglyceridemia or dyslipidemia, respectively. Smokers in the third quartile group revealed increased CVDs risk. The fourth quartile showed significant increased risks in patients suffering from hypercholesterolemia (OR = 1.91 [1.33–2.74]), high LDL-C (OR = 1.88 [1.33–2.66]), and hypertriglyceridemia (OR = 2.31 [1.57–3.41]).

**Conclusions:**

Our findings suggested that assessing hs-CRP is beneficial for predicting CVDs in patients with HTN and DM. Furthermore, specific patients with lipid abnormalities or history of smoking benefits from checking hs-CRP.

## Introduction

Cardiovascular diseases (CVDs) remain the main causes of death worldwide in spite of general health care improvement in both developed and developing countries [1]. By the year 2030, it has been estimated that more than 23.3 million people will die because of cardiovascular complications. Statistics reported that coronary heart disease (CHD) rates will be increased by 160% in middle and low-income countries in Middle East and North Africa. These diseases in which ischemic heart disease (IHD) and stroke are considered the prototypes cause a great economic burden and affect patients’ quality of life. The burden of the CVDs have been predicted to be increased from 47% to 60% by the year 2020. In Europe, annual expenditure for CVDs was estimated to be 169 billion Euro [2,3,4].

Several risk factors including positive family history, hypertension (HTN), type 2 diabetes mellitus (DM), smoking and dyslipidemia have been demonstrated to be responsible in the pathophysiology of CVDs, therefore, they have been categorized as the major traditional CVDs risk factors [5].

Due to inability of above factors in explaining the causality of all cardiovascular events, some new risk factors have been introduced in which inflammatory markers pose the great interest [6,7,8,9]. C-reactive protein (CRP) is a circulatory acute phase reactant produced mainly in the liver, but smooth muscle cells of coronary arteries also make this substance. In addition to its role in pathogenesis of CVDs mostly due to initiation and development of atherosclerotic lesions and causing insulin resistance and type 2 DM, this protein enhances the likelihood of plaque rupture [9,10,11,12]. High sensitivity method are used to measure this acute phase reactant protein, which introduced a novel biomarker named high sensitivity CRP (hs-CRP). This novel biomarker is associated with raising the incidence of cardiovascular events and their major contributing factors including HTN and arterial stiffness as well as atheromatous plaque rupture [8,13,14,15,16,17,18,19,20]. Despite its usefulness, the relationship between this novel marker and CVDs in presence of major CVDs risk factors needs to be identified.

In this study, we aimed to evaluate the predictive role of hs-CRP for cardiovascular events among Iranian population and patients who have diverse CVDs risk factors.

## Methods and Materials

### Study design and population

This study was a nested case-control study in context of a prospective longitudinal population based project called Isfahan cohort study (ICS). This study initiated in three different urban and rural cities of Iran (Isfahan, Najaf Abad and Arak) in 2001 in which the exact methodology has been explained before [21]. ICS is an ongoing study with every 5-year evaluations. From 2^nd^ January till 28^th^ September of 2001, by multi-stage random sampling, anyone aged at least 35 years was eligible for enrollment in the study. A written informed consent form was given to anyone participating in the project. All individuals fulfilled a questionnaire on demographic information, lifestyle behaviors and physical activity status. Then, for each participant, an appointment within the nearest health care clinic was made in order to perform medical interview and physical examinations. Thereafter, telephone surveys were done every two years and after each five years, participants were invited again to health care clinic for assessments exactly like the baseline survey. Incomplete medical data in their documents or unwillingness to continue the project were study exclusion criteria. This study was approved by ethics committee of Isfahan University of Medical Sciences [21].

For this nested case-control study, which was mainly implemented due to financial issues, the case group was chosen from participants in ICS who had fatal, non-fatal myocardial infarction (MI), sudden cardiac death (SCD), fatal and non-fatal stroke and unstable angina (UA) between September 2001 and May 2015. Occurring any of the aforementioned cardiovascular events during the follow-up, put the participants in the case group. On the other hand, control group was selected among healthy individuals without any events with the same duration of attendance in the study and was matched with the case group (density sampling) to make time for risk to be similar between each pair. The controls for each case had the same risk factors at the time of sampling.

### CVDs evaluation

CVDs were categorized as four distinct diseases including MI (fatal and non-fatal), UA, SCD and stroke (fatal and non-fatal). MI was diagnosed as presence of two of the followings: 1) typical chest pain lasting more than 30 minutes, 2) ST segment elevation of more than 0.1 mV in at least two adjacent leads and 3) elevation of cardiac biomarkers troponin I. UA was defined as: typical chest pain lasting at least 20 minutes even at rest or during minimal exercise, changing pattern or new onset of angina concurrent with changes in ST segments or T waves in at least two adjacent electrocardiographic leads. Any deaths outside the hospital, which were occurred in the first 24 hours after starting the symptoms without no other life threatening conditions was defined as SCD. Focal neurological deficit signs and symptoms lasting more than 24 hours of duration were considered as stroke attack.

In a case of death or hospital admission, complementary information was obtained through hospital admission documents and specific checklists. Four cardiologists and one neurologist were two distinct specialties making definitive decisions about four diseases (MI, UA, SCD and stroke). For each participant suffering from any CVDs, a control specimen was selected randomly from individuals with no CVDs but with the same risk factors at the time of event.

### Parameters measurements

Smoking status information was gathered from the questionnaire (yes/no). Body mass index (BMI) was calculated by division of weight in kilograms over squares of height in meters (Kg/m^2^). Blood pressure (BP) was measured using standard cuff and calibrated sphygmomanometer after resting for five minutes. A fasting blood sample of all participants was collected and after serum separation was stored at –70 Celsius (°C) in Isfahan Cardiovascular Research Institute. The following parameters were measured in their blood: fasting blood sugar (FBS), total cholesterol (TC), low density lipoprotein cholesterol (LDL-C), high density lipoprotein cholesterol (HDL-C), triglyceride (TG) and hs-CRP. Hs-CRP was measured using immunoturbidimetric method by Cobas e-411 auto analyzer (Roche Diagnostics International Ltd, Basel, Switzerland) [22]. Other biochemical variables were measured by standard laboratory methods.

Based on BMI status, participants were categorized as overweight (25 kg/m^2^ ≤ BMI < 30 kg/m^2^) or obese (BMI ≥ 30 kg/m^2^). Systolic BP (SBP) and diastolic BP (DBP) of at least 140 and/or 90 millimeter mercury (mmHg), respectively or receiving anti-hypertensive drugs were considered HTN. In terms of lipid indices, the followings were assumed abnormal levels: TC ≥ 200 milligrams per deciliter (mg/dl), LDL-C ≥ 160 mg/dl, HDL-C < 40 mg/dl in men and <50 mg/dl in women and TG ≥ 150 mg/dl. DM was classified as FBS ≥ 126 mg/dl or 2-hour post prandial glucose ≥ 200 mg/dl or being under DM therapy. Metabolic syndrome was assessed using the revised version of the ATP Ш criteria, adjusted by the American Heart Association and National Heart, Lung and Blood Institute (NHLBI) [23,24]. At least three components of this criteria are required to diagnose this condition: 1) FBS ≥ 100 mg/dl or receiving anti-diabetic agents, 2) waist circumference (WC) ≥ 102 centimeter (cm) in men and ≥88 cm in women indicating abdominal obesity, 3) SBP and DBP of at least 130 or 85 mmHg, respectively or being under HTN treatment, 4) TG of at least 150 mg/dl or taking drugs for hypertriglyceridemia and 5) HDL-C of less than 50 and 40 mg/dl for men and women, respectively.

### Statistical analysis

Numerical and categorical values were reported as mean ± standard deviation and number (percentages), respectively. Qualitative and quantitative variables in case and control groups were analyzed using chi-square and t-test, respectively.

Stepwise logistic regression model was used to calculate adjusted odds ratio (OR) in total study population and in individuals with each specific CVDs risk factor including dyslipidemia, DM, HTN, obesity and cigarette smoking via different models. In model 1, age and sex were adjusted. Model 2 was adjusted by the model 1 plus dyslipidemia. Age, sex and HTN were adjusted in the model 3. In addition to adjustment for age and sex, DM and cigarette smoking were adjusted in model 4 and 5, respectively. Age and sex were adjusted in both models 6 and 7 with addition of obesity and metabolic syndrome in the former and latter models, respectively. Finally, all variables (age, sex, dyslipidemia, HTN, DM, obesity and cigarette smoking) were adjusted in the last model. Each risk factor group was analyzed in crude and age plus sex adjusted model. In this analysis, the calculated OR based on hs-CRP as a continuous value in quartiles in total and specific groups with different models were done just like previously mentioned. Hs-CRP quartiles were defined as Q1: 0.1–2.3 mg/l, Q2: 2.4–3 mg/l, Q3: 3.1–4 mg/l and Q4: 4.1–14 mg/l with further analyzing the relation of each CVDs risk factors in each specific hs-CRP quartile in order to perform an additional sensitivity analysis.

All analyses were done using Stata Statistical Software, Release 15.0 (Stata Corporation, College Station, TX, USA). P-values less than 0.05 were considered statistically significant.

## Results

### Baseline characteristics

After mean duration of 128 (68–137.5) months, 502 cardiovascular events were occurred, 83 non-fatal strokes (16.5%), 18 fatal strokes (3.6%), 30 fatal MI (6%), 81 non-fatal MI (16.1%), 220 UA (43.8%) and 70 SCD (14%). This case group was matched with 538 controls with mean follow-up of 72 (45–109) months.

Participants’ baseline characteristics in both groups are provided in Table 1. The case group mean age was 57.7 ± 11 years at the baseline, whereas the control group was 49.2 ± 11 years old (P < 0.05). Males were the dominant sex group in the case group (55.5%). BMI status was not significant between these two groups. Individuals with CVDs history smoked more frequently than ones without any CVDs. In terms of blood pressure indices, both SBP and DBP were higher in case group. HTN was significantly less prevalent among control subjects (24.7% vs. 50%, P = 0.0001). Mean TC, LDL-C, and TG levels were significantly different between two groups. Mean HDL-C level was not statistically different between studied groups. Individuals within case group had a higher hs-CRP levels compared to controls (3.5 ± 1 mg/l vs. 3.2 ± 1 mg/l, P = 0.04). Hypercholesterolemia, high LDL-C and hypertriglyceridemia were more prevalent among the case group. Participants suffering from any CVDs had more frequencies of dyslipidemia, DM and metabolic syndrome. Moreover, this group had significantly higher numbers of cardiovascular risk factors (P = 0.0001).

**Table 1:** Baseline characteristics of participants in case and control group.

Variables		Case group (n = 502)	Control group (n = 538)	*P* value

**Age at baseline (years)**		57.7 ± 11.7	49.28 ± 11.12	0.00001
**Sex (Male) n (%)**		279 (55.58)	251 (46.65)	0.004
**BMI (kg/m^2^)**		27.06 ± 4.49	26.7 ± 4.42	**0.19**
**Overweight, n (%)**		226 (45.02)	218 (40.52)	**0.14**
**Obesity, n (%)**		111 (22.11)	127 (23.61)	**0.56**
**Smoking, n (%)**		132 (26.35)	105 (19.59)	0.01
**SBP (mmHg)**		132.69 ± 24.32	120.71 ± 19.39	0.00001
**DBP (mmHg)**		82.88 ± 12.48	78.38 ± 10.93	0.00001
**Hypertension, n (%)**		251 (50)	133 (24.72)	0.0001
**Cholesterol (mg/dl)**		228.25 ± 53.95	214.97 ± 48.74	0.00001
**Hypercholesterolemia, n (%)**		345 (48.73)	316 (58.74)	0.001
**LDL-C (mg/dl)**		139.4 ± 45.83	130.93 ± 40.6	0.001
**High LDL-C, n (%)**		289 (57.57)	263 (48.88)	0.005
**HDL-C (mg/dl)**		46.63 ± 10.49	47.79 ± 10.57	**0.18**
**Low HDL-C, n (%)**		231 (460.2)	239 (44.42)	**0.6**
**Triglyceride (mg/dl)**		211.07 ± 110.15	182.71 ± 96.3	0.00001
**Hypertriglyceridemia, n (%)**		348 (69.32)	294 (54.65)	0.0001
**Dyslipidemia, n (%)**		465 (92.63)	462 (85.87)	0.0001
**Blood sugar (mg/dl)**		100.46 ± 47.82	86.92 ± 28.81	0.00001
**Diabetes mellitus, n (%)**		119 (23.71)	53 (9.85)	0.0001
**Metabolic syndrome (%)**		238 (47.41)	170 (31.60)	0.0001
**Number of CVD Risk factors n, (%)**	**0**	14 (2.79)	44 (8.21)	0.0001
	**1**	136 (27.15)	252 (47.01)	
	**2**	205 (40.92)	191 (35.63)	
	**>2**	147 (29.28)	51 (9.47)	
**Hs-CRP (mg/l)**		3.51 ± 1.87	3.29 ± 1.6	0.04

N: number of patients, BMI: body mass index, kg/m^2^: kilograms per square meter, SBP: systolic blood pressure, DBP: diastolic blood pressure, mmHg: millimeter mercury, LDL-C: low-density lipoprotein cholesterol, HDL-C: high-density lipoprotein cholesterol, mg/dl: milligrams per deciliter, Hs-CRP: high sensitivity C-reactive protein, mg/l: milligrams per liter.

Data on CVDs prediction based on hs-CRP as a continuous variables and theirs adjustments in studied population were depicted in Table 2. According to the table, before adjustment for different variables, the risk of CVDs was 1.06-fold higher (95% CI: [1.02–1.12]) for each unit increase in hs-CRP (*P* = 0.005). Furthermore, after adjusting for all of the variables, this increased OR was remained intact, with the value of 1.05 (95% CI: [1.003–1.1], *P* = 0.04).

**Table 2:** CVD prediction based on hs-CRP with Logistic regression model in studied participants (n = 1040).

	OR	95% CI	P value

Crude	1.06	1.02–1.12	0.005
Model 1	1.05	1.001–1.1	0.04
Model 2	1.05	1.009–1.11	0.01
Model 3	1.04	0.99–1.09	0.07
Model 4	1.05	1.0005–1.1	0.04
Model 5	1.05	1.004–1.1	0.04
Model 6	1.05	1.007–1.11	0.03
Model 7	1.04	0.99–1.09	0.06
Model 8	1.05	1.003–1.1	0.04

OR: odds ratio, CI: confidence interval. *Model 1:* adjusted for age & sex. *Model 2:* adjusted for age, sex and dyslipidemia. *Model 3:* adjusted for age, sex and hypertension. *Model 4:* adjusted for age, sex and diabetes. *Model 5:* adjusted for age, sex and cigarette smoking. *Model 6:* adjusted for age, sex and obesity. *Model 7:* adjusted for age, sex and Met. Syndrome. *Model 8:* adjusted for age, sex, dyslipidemia, HTN, DM, obesity and cigarette smoking.

CVDs prediction based on hs-CRP levels in patients with each specific CVDs risk factor including dyslipidemia, DM, HTN, obesity and cigarette smoking and their adjustments were depicted in Table 3. According to the table, in patients suffering from dyslipidemia or DM, the OR of CVDs were 1.06 (95% CI: [1.01–1.11]) and 1.14 (95% CI: [1.03–1.26]) folds higher for each unit increase in hs-CRP levels in the crude model, respectively. However, this relationship was not remained significant in model adjusted for age and sex. Furthermore, neither crude nor adjusted models showed no significant association in terms of CVDs OR in smokers or patients with hypertension or obesity.

**Table 3:** CVD prediction based on hs-CRP with Logistic regression model in studied participants with dyslipidemia, DM, HTN, Obesity and cigarette smoking.

Risk factors	Models	OR	95% CI	P value

Dyslipidemia (N = 927)	Crude	1.06	1.01–1.11	0.008
	age & sex adjusted	1.04	0.99–1.10	0.05
Diabetes mellitus (N = 172)	Crude	1.14	1.03–1.26	0.01
	age & sex adjusted	1.08	0.97–1.21	0.1
Hypertension (N = 384)	Crude	1.05	0.99–1.11	0.07
	age & sex adjusted	1.05	0.99–1.12	0.06
Obesity (N = 238)	Crude	1.08	0.99–1.18	0.05
	age & sex adjusted	1.06	0.97–1.16	0.1
Cigarette smoking (N = 237)	Crude	0.97	0.87–1.08	0.6
	age & sex adjusted	0.97	0.87–1.08	0.6

N: number, OR: odds ratio, CI: confidence interval.

Table 4 showed data on OR of CVDs according to different hs-CRP quartiles in our total study population. Although individuals in higher hs-CRP level had increased chances of getting cardiovascular events (OR: 1.36, 95% CI: [1.06–1.74]), last model adjusted for potential confounding variables failed to prove any significant association (OR: 1.26, 95% CI: [0.98–1.62]).

**Table 4:** CVD prediction based on hs-CRP quartiles with Logistic regression model in studied participants (n = 1040).

OR (95% CI)				

	Q1	Q2	Q3	Q4

**Crude**	ref	1.14 (0.89–1.46)	0.97 (0.75–1.25)	1.36 (1.06–1.74)
**Model 1**	ref	1.15 (0.90–1.47)	0.91 (0.71–1.18)	1.23 (0.96–1.58)
**Model 2**	ref	1.16 (0.91–1.49)	0.91 (0.71–1.17)	1.30 (1.01–1.67)
**Model 3**	ref	1.13 (0.88–1.44)	0.91 (0.71–1.19)	1.22 (0.22–1.57)
**Model 4**	ref	1.14 (0.89–1.45)	0.91 (0.71–1.18)	1.21 (0.94–1.55)
**Model 5**	ref	1.15 (0.90–1.47)	0.90 (0.69–1.16)	1.23 (0.96–1.58)
**Model 6**	ref	1.14 (0.89–1.46)	0.91 (0.70–1.18)	1.21 (0.97–1.56)
**Model 7**	ref	1.12 (0.87–1.43)	0.90 (0.69–1.16)	1.22 (0.95–1.57)
**Model 8**	ref	1.14 (0.89–1.46)	0.91 (0.69–1.16)	1.26 (0.98–1.62)

OR: odds ratio, CI: confidence interval, Q1: 0.1–2.3 mg/l, Q2: 2.4–3 mg/l, Q3: 3.1–4 mg/l and Q4: 4.1–14 mg/l, ref: reference. *Model 1:* adjusted for age & sex. *Model 2:* adjusted for age, sex and dyslipidemia. *Model 3:* adjusted for age, sex and hypertension (HTN). *Model 4:* adjusted for age, sex and diabetes mellitus (DM). *Model 5:* adjusted for age, sex and cigarette smoking. *Model 6:* adjusted for age, sex and obesity. *Model 7:* adjusted for age, sex and Metabolic Syndrome. *Model 8:* adjusted for age, sex, dyslipidemia, HTN, DM, obesity and cigarette smoking.

CVDs OR divided individually by the different cardiovascular risk factors and based on the different hs-CRP quartiles and are provided in Table 5. Individuals suffering from dyslipidemia and in the highest quartile (4.1–14 mg/l) had an increased OR of 1.44 (95% CI: [1.11–1.87]) for CVDs comparing to the reference group in crude model. Moreover, this increased chance remained significant in sex and age adjusted model in comparison to participants with dyslipidemia and their hs-CRP levels ranged 0.1–2.3 mg/l. The only significant relation in diabetic patients was found just in crude model in which there was an 82% (OR = 1.82, 95% CI: [1.10–3.00]) increased odds of getting CVDs in individuals within Q4 category compared to subjects with reference hs-CRP levels. Both crude and adjusted models failed to prove any significant relationship in terms of CVDs risk and hs-CRP levels in smokers, hypertensive or obese patients.

**Table 5:** Association between CDV and hs-CRP quartiles in Isfahan Cohort with Logistic regression model in patients with dyslipidemia, DM, HTN, obesity and cigarette smoking.

Risk factors	Models	OR (95% CI)			

		Q1	Q2	Q3	Q4

Dyslipidemia (N = 927)	Crude	ref	1.23 (0.95–1.58)	1.00 (0.76–1.30)	1.44 (1.11–1.87)
	age & sex adjusted	ref	1.28 (0.99–1.65)	0.94 (0.72–1.23)	1.31 (1.01–1.70)
Diabetes mellitus (N = 172)	Crude	ref	1.16 (0.70–1.95)	0.93 (0.53–1.63)	1.82 (1.10–3.00)
	age & sex adjusted	ref	1.14 (0.68–1.91)	0.83 (0.47–1.45)	1.33 (0.80–2.23)
Hypertension (N = 384)	Crude	ref	0.92 (0.64–1.31)	0.90 (0.63–1.28)	1.18 (0.83–1.68)
	age & sex adjusted	ref	0.93 (0.65–1.33)	0.92 (0.64–1.32)	1.13 (0.80–1.60)
Obesity (N = 238)	Crude	ref	1.04 (0.61–1.78)	1.10 (0.64–1.89)	1.38 (0.81–2.37)
	age & sex adjusted	ref	1.07 (0.62–1.83)	1.19 (0.69–2.04)	1.22 (0.71–2.10)
Cigarette smoking (N = 237)	Crude	ref	1.17 (0.72–1.91)	1.02 (0.63–1.65)	0.98 (0.58–1.66)
	age & sex adjusted	ref	1.27 (0.78–2.07)	1.06 (0.66–1.71)	1.01 (0.60–1.70)

Q1: 0.1–2.3 mg/l, Q2: 2.4–3 mg/l, Q3: 3.1–4 mg/l and Q4: 4.1–14 mg/l.

Table 6 provides information on OR of multiple variables across different hs-CRP quartiles. Despite increased OR of CVDs occurrence within the first and second hs-CRP quartiles in males compared with females, this trend was not significant within the next two quartiles (Q3 and Q4). Increasing age was associated with raising ORs of probable CVDs in a way that in all four quartiles, participants who were aged 45–55 or more than 55 years old, had increased risks of getting these diseases compared with subjects less than 45 years. Overweight and obesity as well as HDL-C level failed to show any significant connections in terms of cardiovascular events within hs-CRP quartiles. The only significant association in terms of smoking was that smokers in the third quartile had 1.6 times (95% CI: [1.08–2.35], P = 0.01) increased odds of getting CVDs compared to non-smokers. All participants in all hs-CRP categories who had HTN and DM were posed to an increased chance of CVDs incidence. In comparison with subjects having normal levels of cholesterol and LDL-C levels, this increasing pattern was only significant in the highest hs-CRP quartiles showing that individuals suffering from hypercholesterolemia and high LDL-C levels, had greater chance of getting any CVDs (OR: 1.91, 95% CI: [1.33–2.74], P = 0.0001 and OR: 1–88, 95% CI: [1.33–2.66], P = 0.0001, respectively). Individuals suffering from hypertriglyceridemia had 1.93 (95% CI: [1.33–2.81]) and 2.31 (95% CI: [1.57–3.41]) times increased odds within Q2 and Q4 hs-CRP levels, respectively. Furthermore, dyslipidemia within hs-CRP ranges of 2.4–3 mg/l was associated with 3.34 times (95% CI: [1.36–8.17], P = 0.008) greater chance of getting CVDs. Any participants who had more than two CVDs risk factors, had a higher chance of adverse cardiac events in all quartiles compared with healthy subjects.

**Table 6:** CVD prediction in participants’ subgroups based on hs-CRP quartiles.

Variables		hs-CRP quartiles							

		Q1		Q2		Q3		Q4	

*Hs-CRP*	Range (mg/l)	0.1–2.3		2.4–3		3.1–4		4.1–14	
	Mean ± SD	1.84 ± 0.45		2.72 ± 0.19		3.47 ± 0.29		5.75 ± 2	
	n	261		276		261		242	
**Subgroups**		**OR** **(95% CI)**	**P value**	**OR** **(95% CI)**	**P value**	**OR** **(95% CI)**	**P value**	**OR** **(95% CI)**	**P value**

*Gender*	Female	Ref	0.02	Ref	0.04	Ref	0.3	Ref	0.24
	Male	1.52 (1.07–2.18)		1.41 (1.00–1.98)		1.21 (0.83–1.75)		1.22 (0.87–1.73)	
*Age*	<45	Ref		Ref		Ref		Ref	
	45–55	2.41 (1.41–4.12)	0.001	2.39 (1.42–4.03)	0.001	2.64 (1.49–4.69)	0.001	2.56 (1.45–4.51)	0.001
	>55	4.27 (2.63–6.49)	0.0001	3.41 (2.22–5.22)	0.0001	3.72 (2.19–6.32)	0.0001	4.18 (2.55–6.85)	0.0001
*Overweight*	No	Ref	0.73	Ref	0.85	Ref	0.44	Ref	0.35
	Yes	1.06 (0.7–1.51)		1.03 (0.73–1.46)		1.15 (0.79–1.66)		1.17 (0.83–1.65)	
*Obesity*	No	Ref	0.71	Ref	0.39	Ref	0.69	Ref	0.91
	Yes	0.92 (0.59–1.42)		0.83 (0.55–1.26)		1.08 (0.70–1.66)		0.97 (0.64–1.47)	
*Smoking*	No	Ref	0.09	Ref	0.05	Ref	0.01	Ref	0.74
	Yes	1.42 (0.94–2.15)		1.44 (0.98–2.12)		1.6 (1.08–2.35)		0.93 (0.61–1.42)	
*Hypertension*	No	Ref	0.0001	Ref	0.001	Ref	0.0001	Ref	0.0001
	Yes	2.69 (1.89–3.84)		1.83 (1.30–2.57)		2.53 (1.75–3.67)		2.19 (1.55–3.10)	
*Hypercholesterolemia*	No	Ref	0.98	Ref	0.06	Ref	0.12	Ref	0.0001
	Yes	0.99 (0.68–1.44)		1.44 (0.98–2.11)		1.37 (0.91–2.05)		1.91 (1.33–2.74)	
*High LDL-C*	No	Ref	0.63	Ref	0.17	Ref	0.05	Ref	0.0001
	Yes	0.91 (0.64–1.30)		1.27 (0.89–1.82)		1.44 (0.99–2.10)		1.88 (1.33–2.66)	
*Low HDL-C*	No	Ref	0.42	Ref	0.79	Ref	0.31	Ref	0.96
	Yes	0.86 (0.60–1.23)		1.04 (0.74–1.47)		1.20 (0.83–1.74)		1.00 (0.71–1.42)	
*Hypertriglyceridemia*	No	Ref	0.82	Ref	0.001	Ref	0.07	Ref	0.0001
	Yes	0.95 (0.66–1.37)		1.93 (1.33–2.81)		1.44 (0.95–2.18)		2.31 (1.57–3.41)	
*Dyslipidemia*	No	Ref	0.71	Ref	0.008	Ref	0.19	Ref	0.07
	Yes	1.12 (0.58–2.15)		3.34 (1.36–8.17)		1.60 (0.78–3.28)		1.66 (0.95–2.89)	
*Diabetes mellitus*	No	Ref	0.01	Ref	0.008	Ref	0.03	Ref	0.0001
	Yes	1.73 (1.13–2.66)		1.71 (1.15–2.55)		1.62 (1.02–2.57)		2.31 (1.57–3.39)	
*Number of CVD risk factors*	0	Ref		Ref		Ref		Ref	
	1	0.98 (0.34–2.79)	0.98	1.56 (0.47–5.10)	0.46	2.39 (0.57–10.05)	0.23	1.61 (0.64–4.08)	0.30
	2	2.25 (0.81–6.23)	0.11	3.17 (0.99–10.09)	0.05	3.50 (0.85–14.42)	0.08	2.10 (0.82–5.35)	0.12
	>2	3.09 (1.09–8.75)	0.03	5.66 (1.73–18.50)	0.004	7.47 (1.79–31.09)	0.006	4.08 (1.62–10.31)	0.003

SD: standard deviation, n: number, ref: reference, OR: odds ratio, CI: confidence interval, Hs-CRP: high sensitivity C-reactive protein, mg/l: milligrams per liter, LDL-C: low density lipoprotein cholesterol, HDL-C: high density lipoprotein cholesterol.

## Discussion

The purpose of this study was to investigate the predicting role of hs-CRP values for different CVDs in different categories of CVDs risk factors as well as evaluating the association of each CVDs risk factor with each hs-CRP quartiles. Although our findings did not reveal any significant relationship based on OR of CVDs in different categories of hs-CRP levels in each major cardiovascular risk factors including dyslipidemia, DM, HTN, obesity and cigarette smoking, further analysis on each risk factor in each quartile demonstrated that patients with traditional cardiovascular risk factors including HTN, DM, smoking and abnormal lipid profiles had significantly greater chance of suffering from these disorders in the future based on different hs-CRP quartiles, but this relationship was not significant for obesity or being overweight.

This biomarker was statistically independent in predicting specific CVDs, therefore, early diagnosis and management of this potential cardiovascular risk factor will help decreasing the occurrence of cardiovascular events as well as improving economic burden and could be categorized as a prognostic tool in individuals with different CVDs risk factors.

Several studies were in agreement with our findings. For instance, Ebrahimi et al. [25] performed a cross-sectional study in order to evaluate the relationship between hs-CRP levels in presence of CVDs risk factors including DM, HTN and obesity. They recruited 7762 Iranian individuals with no previous history of cardiovascular events, malignancy or inflammatory disorders. They found that hs-CRP > 3 mg/l was associated with higher prevalence of abnormal TC, LDL-C as well as SBP and DBP in comparison with subjects having hs-CRP < 3mg/l. Furthermore, in another observational study, the associations of hs-CRP with CVDs risk factors were evaluated in 8389 Chinese people aged 35 to 64 years old. They observed that this biomarker levels of more than 1.80 mg/l had been positively associated with increasing age, BP and glucose levels. Moreover, 63% of their participants simultaneously suffered from HTN, DM, hypercholesterolemia and obesity had higher levels of hs-CRP, mean of at least 1.80 mg/l [26].

Our data suggested that smokers in the third hs-CRP quartile had increased chance of getting CVDs. This was consistent with other studies demonstrated a positive correlation between smoking and hs-CRP levels [27,28]. Results of Hung et al. [28] study showed that in comparison with non-smokers, anyone who smokes in middle or high levels of hs-CRP had increased odds of coronary vasospasm and this finding suggested that smokers developed vasospasm at lower hs-CRP levels. Another cross-sectional study revealed that all aspects of smoking habits had significant association with hs-CRP biomarker [27]. Since it was proved that hs-CRP levels would be increased in smokers, several health care strategies should be done to decrease the prevalence of smoking in order to decline these two independent cardiovascular risk factors.

Although in several studies a positive relationship was observed between BMI status and hs-CRP levels, our findings were not significant [29,30,31,32,33]. Possible explanation for this strange result might be malnutrition-inflammation syndrome. Due to inflammation responses in the body, and especially in acute phase responses, anorexia and promotion of catabolic process stimulating protein degradation would occur, which consequently declining BMI levels and CVDs risks. Furthermore, nature of the inflammation creates defense against injurious stimuli, therefore, mild elevation in inflammatory biomarkers specifically hs-CRP, which are mostly observed in overweight persons, have another explanation [32]. Several longstanding comprehensive studies are necessary to investigate the exact associations of body weight with hs-CRP values in order to predict the CVDs likelihood.

This study revealed that in all hs-CRP quartiles, hypertensive patients had increased chance of getting CVDs compared to normotensive ones. This finding was in favor of multiple papers acknowledged affirmative connections between these two variables [11,34,35]. A five-year follow-up on 452 Korean individuals revealed that increasing hs-CRP tertiles were positively associated with more HTN rates, independently from the blood pressure [11]. Another study showed that individuals in fourth hs-CRP quartiles had significantly higher blood pressure levels compared with the first one. Moreover, 10-year congestive heart disease risk of hypertensive individuals announced that high hs-CRP values were associated with greater chance of getting the diseases [35]. Several hypothesis have been postulated defining this connections. CRP was reported to be effective on endothelial dysfunction in a way that high levels may reduce nitric oxide production, increase endothelin 1, and up-regulate angiotensin type 1 receptor, therefore, influencing on renin-angiotensin-aldosterone system. On the other hand inflammation mainly due to CRP level is contributed to cause HTN by reducing vessel elasticity and increasing its stiffness [11]. Health care policy makers should perform appropriate programs declining these two common risk factors.

Our data suggested that any diabetic participants in each hs-CRP quartiles had greater chance of CVDs compared to non-diabetics. Positive association between hs-CRP and DM was established in different articles [12,36,37]. Nabipour et al. [12] reported that among 1754 individuals, those suffered from DM had greater levels of CRP compared to people without the disease. DM had a significant OR of 2.03 (95% CI: [1.38–2.98], P < 0.0001) with elevated CRP levels after adjusting for all of the confounding variables [12]. A cohort study with mean 6-year follow-up on 4213 individuals revealed that there was a significant OR of DM incidence across different hs-CRP quartiles and their findings suggested hs-CRP could be an independent variable prompting on DM incidence [36]. Another study demonstrated that hs-CRP was a significant predictor of type 2 macrovascular complications [37]. Genetic factors, increased oxidative stress, dysregulated immune system, direct effects of hs-CRP on vessel walls, and destructing pancreatic β cells are some proposed potential mechanisms [36].

Participants with disturbed lipid indices had increased risks of CVDs incidence mostly within the last hs-CRP quartile except HDL-C levels, which were not significant at all. The associations between lipid profiles and hs-CRP values have been announced to be controversial. For instance, a prospective longitudinal study on at least 27000 healthy women intended to investigate connections of hs-CRP and LDL-C levels with cardiovascular outcomes demonstrated that abnormal levels of each variable were associated with greater risks of CVDs; however, these two variables had minimal correlation with each other and was suggested that each of those biomarkers detected separate high risk group [8]. On the other hand, Whelton et al. [38] performed a cohort study on 8947 participants and revealed that the associations in terms of TC and LDL-C was significant among individuals less than 65 years, but borderline and insignificant results were achieved among subjects aged at least 65 years with TC and LDL-C, respectively. Due to different sample size, study designs, and various study conduction places, several multidisciplinary projects must have been done to investigate the exact relationships.

Quite reasonable sample size and a good time period follow-up for occurrence of CVDs were some advantages of our study, but this present study was not free of limitations. The retrospective design of project may influence on outcomes and caution must be taken out in case of generalization the results. Furthermore, we did not collect the relevant data about medications used by each individual. Consequently, this problem could affect our results in terms of probable influence on CVDs occurrence during the follow-up duration in addition to their possible effects in hs-CRP levels. Lack of information about the possible effect of psychological factors, especially stress, which was previously approved to be determining factor in CVDs incidence might be considered another limitation of the current study. Further studies with prospective design are warranted to determine the usefulness of hs-CRP measurement in different groups.

## Conclusion

In this study we determined the predicting role of hs-CRP levels for CVDs in different subgroups of Iranian population. We observed that this biomarker is associated with higher odds of CVDs. Hypertensive and diabetic patients had the greatest benefit of hs-CRP measurement for CVDs prediction. Additionally, specific patients with lipid abnormalities or history of smoking can benefit from checking the hs-CRP.

## Abbreviations

CVD: Cardiovascular diseases

CHD: coronary heart disease

IHD: ischemic heart disease

HTN: hypertension

DM: type 2 diabetes mellitus

CRP: C – reactive protein

hs-CRP: high sensitivity C – reactive protein

ICS: Isfahan cohort study

MI: Myocardial infarction

SCD: Sudden cardiac death

UA: Unstable angina

mV: millivoltage

mg: milligrams

dl: deciliter

l: liter

mmHg: millimeter mercury

cm: centimeter

BMI: Body mass index

Kg/m^2^: Kilograms per square meter

BP: Blood pressure

SBP: systolic blood pressure

DBP: diastolic blood pressure

FBS: Fasting blood sugar

TC: Total cholesterol

LDL-C: Low density lipoprotein cholesterol

HDL-C: High density lipoprotein cholesterol

TG: triglyceride

WC: waist circumference

OR: odds ratio

## Author Information

Pooya Koosha is first author and Hamidreza Roohafza is co- first author.
